# Supplementary material for: Forecasting influenza hemagglutinin mutations through the lens of anomaly detection
Source: Sci Rep. 2023 Sep 11;13:14944. doi: 10.1038/s41598-023-42089-y (PMC10495359; doi:10.1038/s41598-023-42089-y)
Supplement: Supplementary file 1 — Supplementary Information. [file 41598_2023_42089_MOESM1_ESM.pdf]

Supplementary Materials

Figures

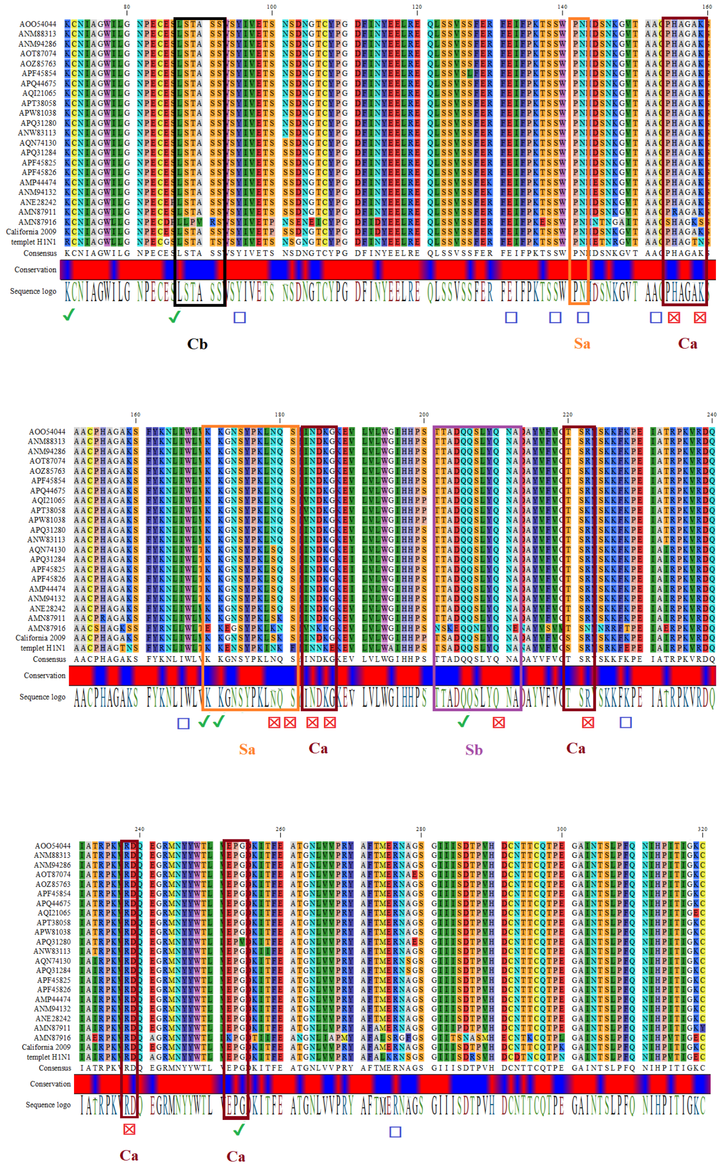

Figure S1. The antigenic structure and the proposed mutations by the current study for H1N1. Symbols represents the proposed alteration by the model in right location (✓), not detected important alteration in antigenic site (☒), and proposed alteration by model that does not reflect any important or high prevalence alteration (☐).

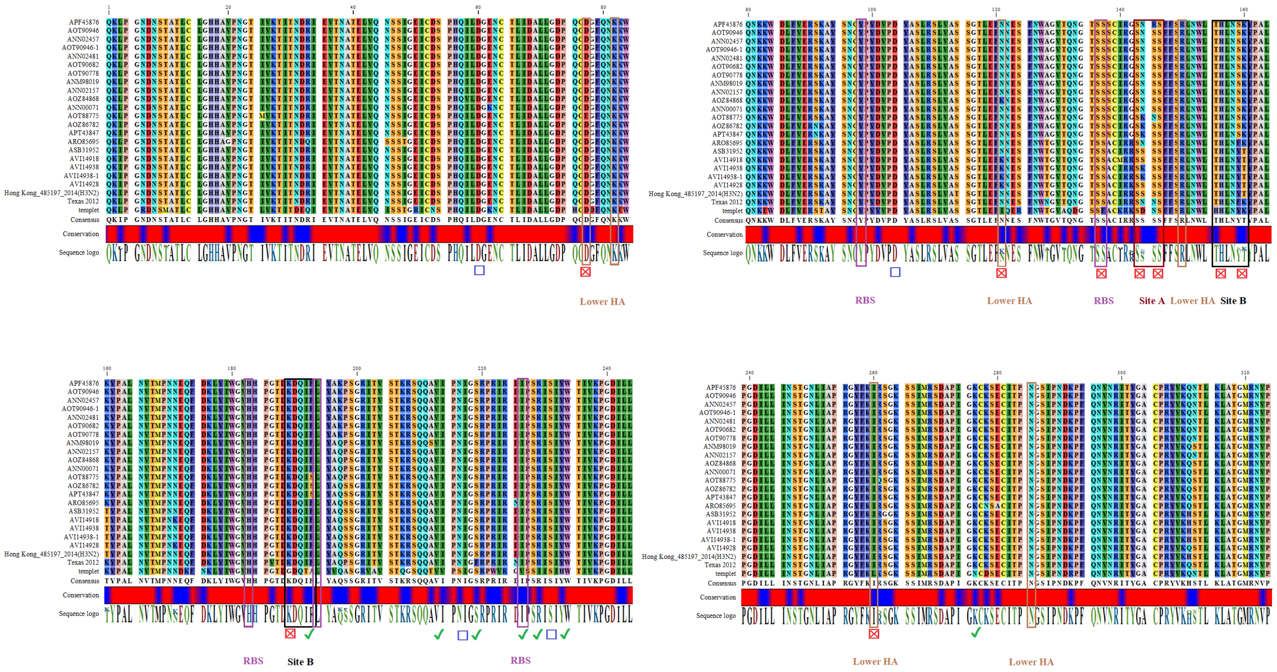

Figure S2. The antigenic structure and the proposed mutations by the current study for H3N2. Alignment numbers are set based on the HA gene without the first 16 amino acids of the signal peptide in the figure for illustration of the antigenic locations. While in the assessment by the algorithm, we evaluate this 16 amino acids and represented numbers for locations in table S1.

Symbols represent the proposed alteration by the model in right location (✓), not detected important alteration in antigenic site (☒), and proposed alteration by the model that does not reflect any important or high prevalence alteration (☐).

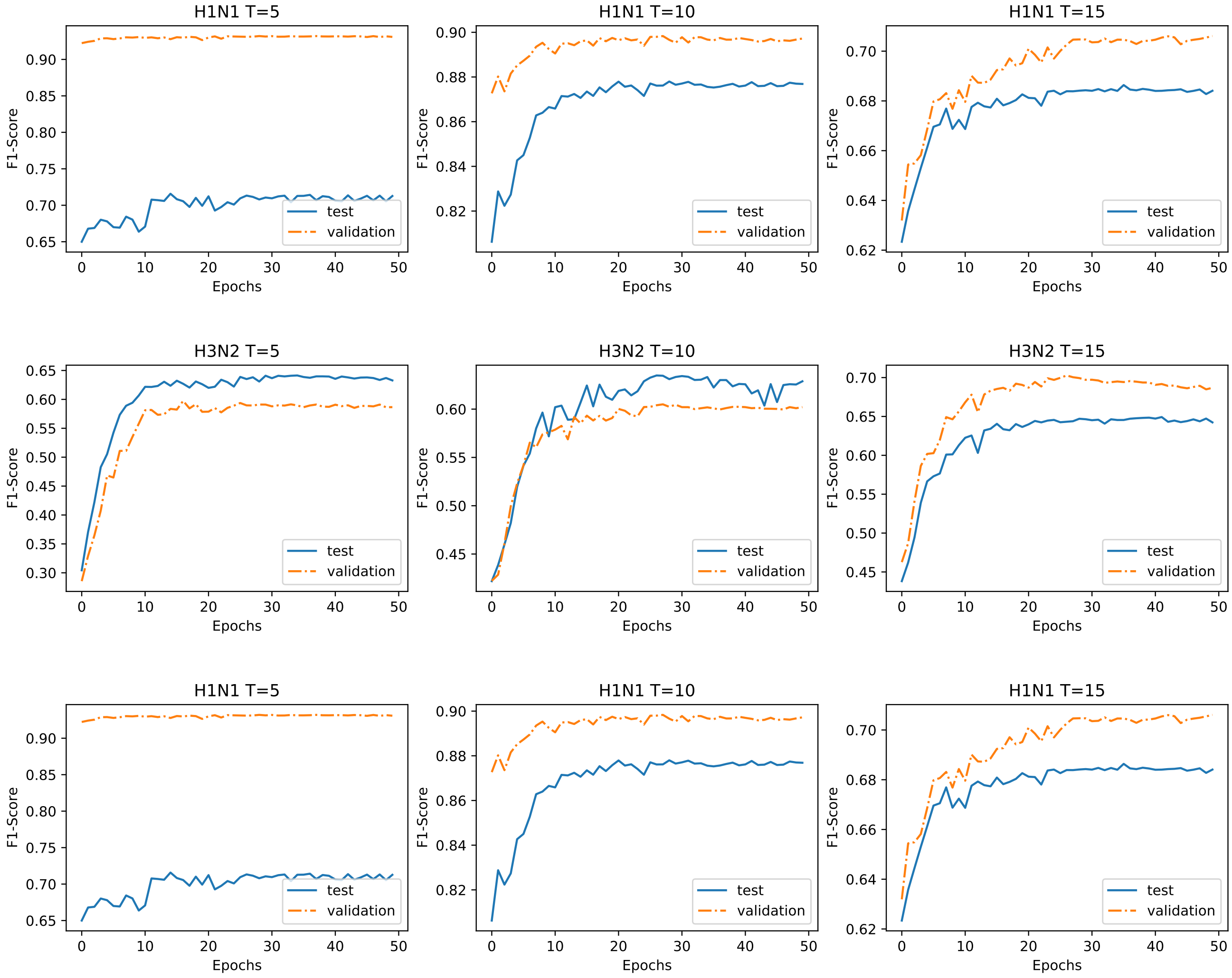

Figure S3. F1-Score of each experiment over epochs. The validation F1-Score in every epoch is achieved by adjusting the threshold in such a way that maximizes this score on the validation data. Later in the same epoch, this threshold is applied to the test dataset to get the test F1-Score.

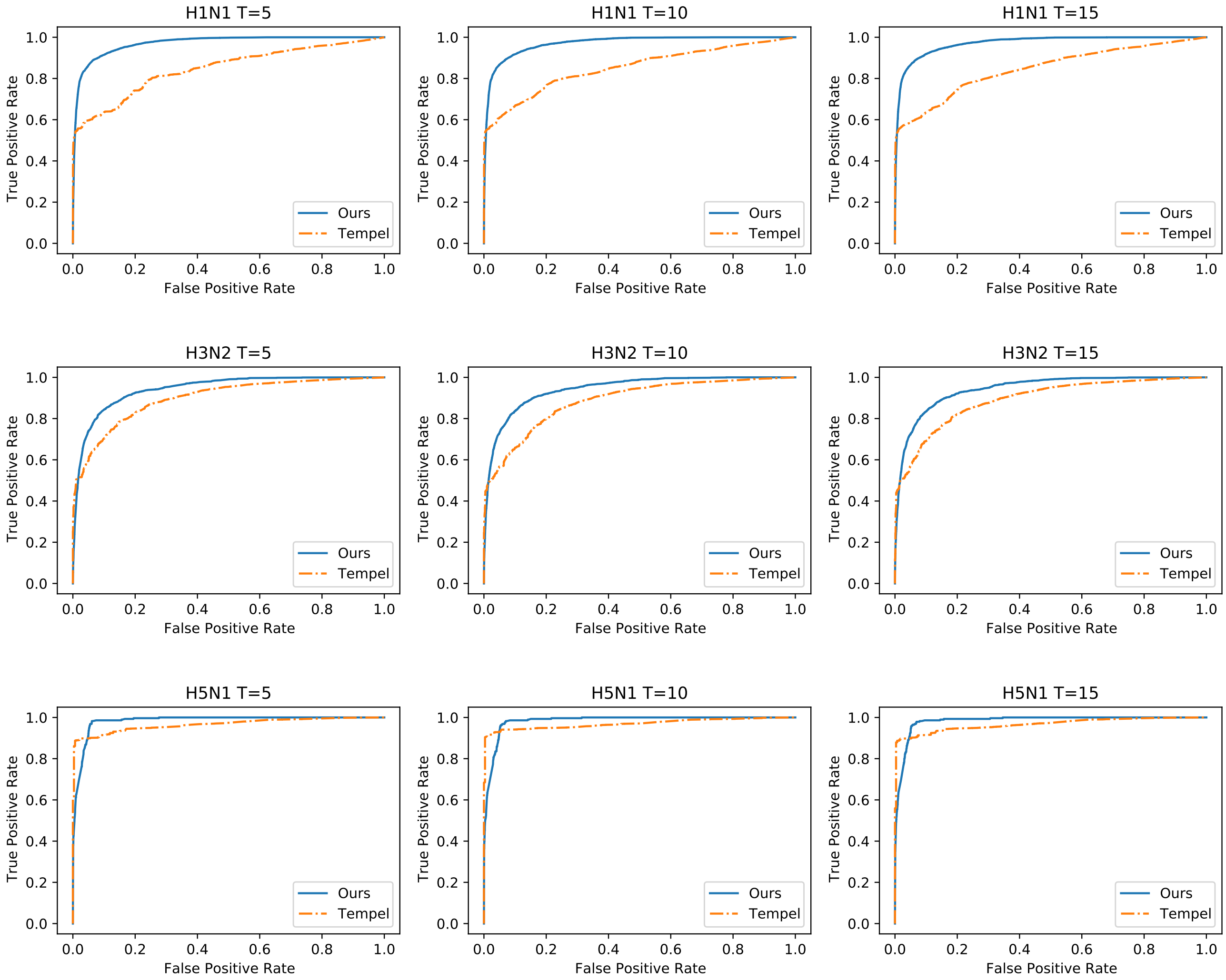

Figure S4. The ROC curves of our model and Tempel for each experiment on the HA dataset.

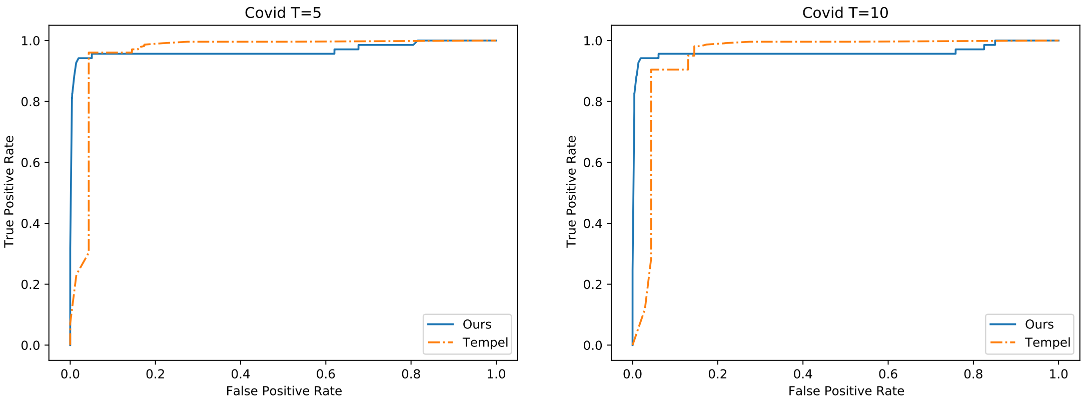

Figure S5. The ROC curves of our model and Tempel for each experiment on the SARS-CoV-2 dataset.

Tables

Table S1. The model's results for different possible amino acid alteration locations.

Virus type	Mutation	Current study			Tempel		
		Mutation Recall	Mutation Precision	Mutation*	Mutation Recall	Mutation Precision	Mutation*
H1N1(T5, T10, T15)	69	0.92	0.97	Y	0.95	0.97	Y
	70	0.93	0.95	N	0.9	0.95	N
	85	0.89	0.95	Y	0.89	0.97	Y
	95	0.97	1	N	0.97	1	N
	132	0.93	0.89	N	0.95	0.9	N
	139	0.89	0.95	N	0.95	1	N
	140	0.91	0.91	N	0.89	0.91	N
	142	0.92	1	N	0.92	1	N
	152	0.91	1	Y	0.93	1	N
	165	-	-	-	0.95	0.97	Y
	166	0.84	0.97	N	-	-	-
	168	0.97	0.95	N	0.9	0.95	N
	170	0.88	0.97	Y	0.88	0.97	Y
	174	-	-	-	0.9	0.92	Y
	205	0.95	0.93	Y	0.93	0.93	Y
	226	0.9	0.97	Y	0.89	0.95	Y
	253	0.93	0.97	Y	0.93	0.97	Y
	276	0.86	0.97	Y	0.9	0.97	Y
H3N2(T5, T10, T15)	44	0.75	0.5	N	1	1	N
	88	-	-	-	0.84	1	N
	177	1	0.83	N	-	-	-
	193	1	0.85	Y	-	-	-
	197	1	0.85	Y	0.5	1	Y
	198	0.58	0.31	Y	0.58	0.77	Y
	201	0.5	0.28	N	0.25	0.5	N
	203	0.66	0.77	N	0.41	1	N
	207	1	0.8	N	0.5	1	N
	208	0.87	0.85	N	0.57	1	N
	209	0.75	0.6	N	0.5	1	N
	212	0.45	0.62	Y	0.36	1	Y
	213	0.46	0.8	N	0.28	1	N
	214	0.57	0.75	N	0.3	1	N
	215	0.8	0.5	N	0.4	0.67	N
	216	0.69	0.5	N	0.53	1	N
	217	0.9	0.76	N	0.81	1	N
	218	0.95	0.87	N	0.45	1	N
H5N1(T5, T10, T15)	82	1	1	Y	1	1	Y
	91	1	1	N	1	1	N
	116	1	1	Y	1	1	Y
	118	-	-	-	1	1	N
	130	-	-	-	1	1	Y
	152	1	1	Y	1	1	Y
	162	1	1	N	1	1	N
	163	1	1	N	1	1	N
	165	1	0.5	N	-	-	-
	166	1	1	Y	1	1	Y
	179	0.5	1	Y	-	-	-
	172	0.5	1	Y	-	-	-
	182	0.25	0.66	N	0.25	0.67	N
	185	1	1	Y	0.5	1	Y
	193	1	1	N	1	1	N
	205	1	1	Y	1	1	Y
	242	1	1	Y	1	1	Y

Y: Yes, N: No, *: No, does not express that there is no mutation in all of the available data for 2016. Only that the mutations are not present in the current random sampling.
